# Structures of *C*-mannosylated anti-adhesives bound to the type 1 fimbrial FimH adhesin

**DOI:** 10.1107/S2052252516002487

**Published:** 2016-02-26

**Authors:** Jerome de Ruyck, Marc F. Lensink, Julie Bouckaert

**Affiliations:** a Université Lille, CNRS, UMR 8576–UGSF–Unité de Glycobiologie Structurale et Fonctionnelle, 59000 Lille, France

**Keywords:** bacterial adhesion, anti-adhesives, *C*-mannosides, FimH, variable immunoglobulin fold, protein structure, X-ray crystallography, intermolecular interactions, hydrogen bonding

## Abstract

Selective inhibitors of the type 1 fimbrial adhesin FimH are recognized as attractive alternatives for antibiotic therapies and prophylaxes against *Escherichia coli* infections such as urinary-tract infections. To construct these inhibitors, the α-d-mannopyranoside of high-mannose *N*-glycans, recognized with exclusive specificity on glycoprotein receptors by FimH, forms the basal structure. A hydrophobic aglycon is then linked to the mannose by the O1 oxygen inherently present in the α-anomeric configuration. Substitution of this O atom by a carbon introduces a *C*-glycosidic bond, which may enhance the therapeutic potential of such compounds owing to the inability of enzymes to degrade *C*-glycosidic bonds. Here, the first crystal structures of the *E. coli* FimH adhesin in complex with *C*-glycosidically linked mannopyranosides are presented. These findings explain the role of the spacer in positioning biphenyl ligands for interactions by means of aromatic stacking in the tyrosine gate of FimH and how the normally hydrated *C*-glycosidic link is tolerated. As these new compounds can bind FimH, it can be assumed that they have the potential to serve as potent new antagonists of FimH, paving the way for the design of a new family of anti-adhesive compounds against urinary-tract infections.

## Abstract 2

A comparison of the different conformations adopted by *C*-glycosylated mannosides in FimH crystal structures is reported together with the investigation of new interactions.

## 1. Introduction   

**Figure 1:**
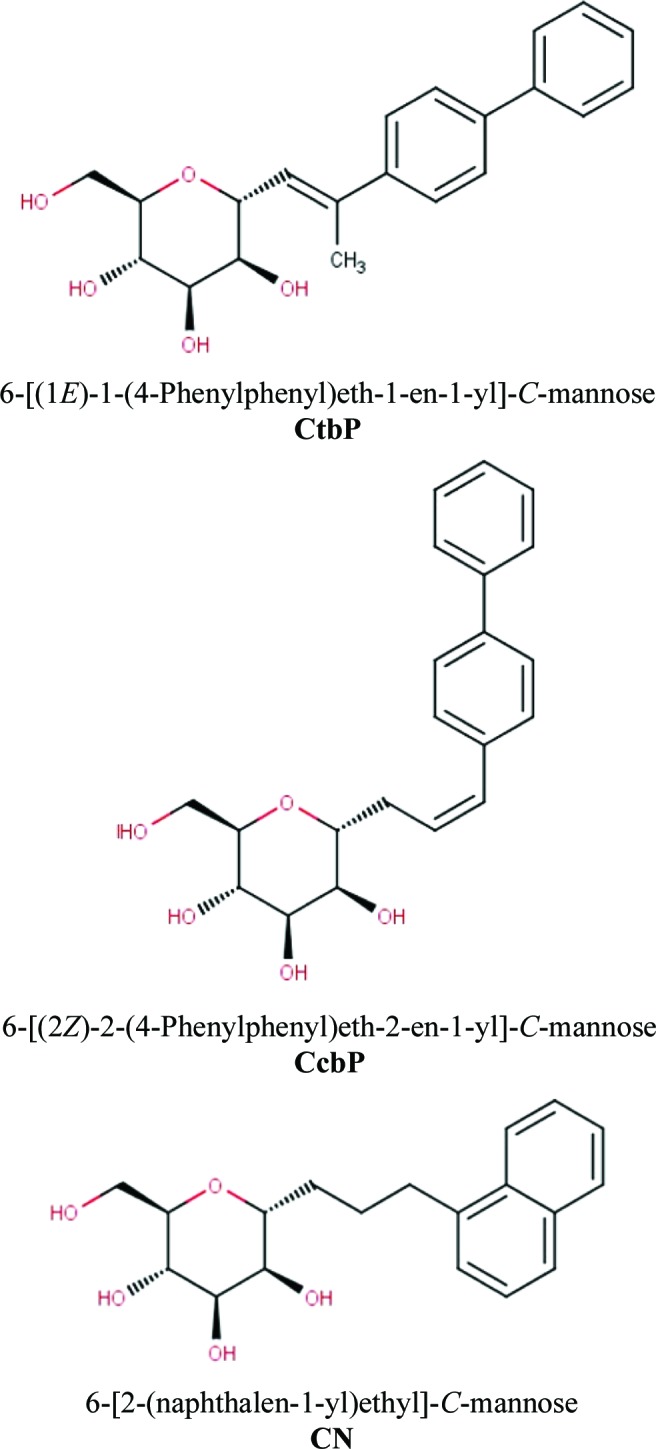
Chemical structures of the studied *C*-glycosidically linked α-d-mannopyranosides.

Urinary-tract infections (UTIs) are some of the most common infections, affecting millions of people each year, especially women (Nicolle, 2008 ▸). The large majority of UTIs are caused by uropathogenic *Escherichia coli* (UPEC) that are able to invade the urothelial cells in the bladder, form biofilms and cause recurrent infections (Wiles *et al.*, 2008 ▸). The adherence of UPEC to the urothelial surface is mediated through the mannose-specific lectin FimH, the natural ligand of which is the mannosylated glycoprotein uroplakin Ia present on urothelial cells (Mulvey, 2002 ▸). Mannose-based FimH antagonists compete with this interaction and prevent bacterial adhesion and hence infection (Nagahori *et al.*, 2002 ▸). Rational drug design based on X-ray crystallographic structures of FimH bound to various α-d-mannosides has provided a wide range of *O*- or *N*-mannosylated inhibitors bearing hydrophobic aglycons (Brument *et al.*, 2013 ▸; Fiege *et al.*, 2015 ▸; Han *et al.*, 2010 ▸; Klein *et al.*, 2010 ▸; Roos *et al.*, 2013 ▸; Wellens *et al.*, 2008 ▸; Cusumano *et al.*, 2011 ▸). Biaryl *O*-linked mannosidic compounds have been evaluated using ITC as nanomolar inhibitors with inhibition constants in the range 1–20 n*M* (Fiege *et al.*, 2015 ▸). In each case the mannose moiety is tightly bound and is involved in an extended hydrogen-bonding network, while the hydrophobic moiety interacts with the two tyrosines (Tyr48 and Tyr137) at the entrance to the FimH-binding domain. Changing the O atom to a carbon in the inhibitors, is of greater interest for therapeutic purposes as enzymes are less able to degrade *C*-glycosidic bonds. Moreover, biaryl *C*-linked mannosidic compounds similar to our compounds have been confirmed to be nanomolar inhibitors of FimH (Mydock-McGrane *et al.*, 2015 ▸). However, structural information on the effect of the change of the nature of the atom making the glycosidic linkage between the α-d-mannose and the biaryl group has not been available to date. Here, the first crystal structures of *C*-glycosidically linked α-d-mannopyranosides in complex with the FimH adhesin of uropathogenic *E. coli* J96 are reported. Two ligands are decorated with a *para*-biphenyl group on a *C*-linkage to an ethene spacer (CcbP) or to an ethene with a branched methyl spacer (CtbP) and one with a naphthyl group on a *C*-linkage to an ethyl spacer (CN) (Fig. 1 ▸).

## 2. Methods   

### 2.1. Expression and purification of recombinant FimH   

The receptor-binding domain (RBD) of the FimH protein (residues 1–158) of UPEC J96 was expressed from plasmid pMMB91 transformed into *E. coli* C43 (DE3) cells and purified according to a previously described protocol (Wellens *et al.*, 2008 ▸).

### 2.2. Crystallization   

Ligands were dissolved in 50% DMSO to a stock concentration of 200 m*M*. Complete names and structures are reported in Fig. 1 ▸. The sitting-drop vapour-diffusion method was used to co-crystallize FimH (18 mg ml^−1^ in 20 m*M* HEPES pH 7.4, 150 m*M* NaCl) with CtbP (final concentration 2 m*M*) at 20°C. The crystals were equilibrated against a well containing 30%(*v*/*v*) 2-propanol, 100 m*M* Tris–HCl pH 8.5, 200 m*M* ammonium acetate. The complexes with CcbP and CN were obtained by soaking ligand-free FimH crystals grown in 1 *M* Li_2_SO_4_, 100 m*M* Tris pH 8.6, 10 m*M* NiCl_2_, 0.2 *M* nondetergent sulfobetaine 201, overnight with a ligand concentration of 5 m*M*.

### 2.3. Data collection and structure determination   

Prior to data collection, crystals were cryoprotected using the crystallization solution complemented with 20% ethylene glycol for CtbP or with 20% glycerol for CcbP and CN, and were flash-cooled in liquid nitrogen. X-ray diffraction data were collected at 100 K on the PROXIMA1 beamline at the SOLEIL synchrotron (Gif-sur-Yvette, France) to resolutions of 1.30, 2.45 and 2.40 Å for FimH–CtbP, FimH–CcbP and FimH–CN, respectively. The data sets were recorded on a Pilatus 6M detector (Dectris) and processed using *XDS* (Kabsch, 2010 ▸). Phasing was performed by molecular replacement (*Phaser*; McCoy *et al.*, 2007 ▸) using chain *A* of the apo FimH lectin domain (PDB entry 4auu; Wellens *et al.*, 2012 ▸). All water and ligand molecules were removed from the search structure. Rebuilding of the initial model using *AutoBuild* (Terwilliger *et al.*, 2008 ▸) was then performed. Subsequently, all-atom isotropic temperature-factor refinement cycles were performed with *phenix.refine* (Afonine *et al.*, 2012 ▸). Electron-density maps were inspected using *Coot* (Emsley *et al.*, 2010 ▸) and the quality of the model was analyzed using *MolProbity* (Chen *et al.*, 2010 ▸). Data-collection and refinement statistics are presented in Table 1 ▸. *PyMOL* was used to generate high-quality images of the structure.

## 3. Results   

### 3.1. Crystal structure of FimH–CtbP   

**Figure 2:**
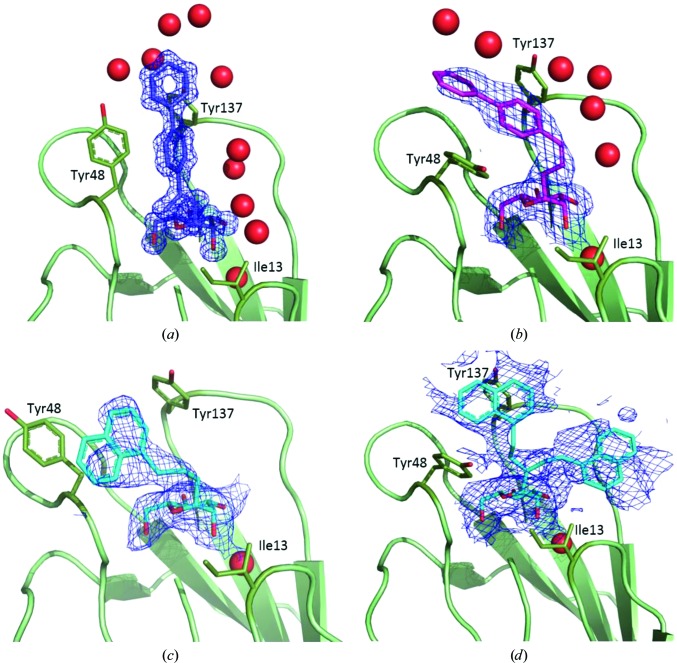
FimH complexes and representations of the 2*mF*_o_ − *DF*_c_ electron-density maps for the ligands (*a*) CtbP bound in the closed tyrosine gate with CtbP contoured at 2.0σ, (*b*) CcbP bound in the open tyrosine gate of FimH contoured at 1.0σ, (*c*) CN (chain *B*) bound in the half-open tyrosine gate and contoured at 1.0σ and (*d*) CN (chain *A*) bound in the open tyrosine gate contoured at 1.0σ. In the latter, the naphthyl group cannot be stabilized and we observed a continuum of electron density for the ligand from Ile13 to Tyr48.

Co-crystallization of FimH with CtbP yielded an ortho­rhombic crystal form that was distinct from that of ligand-free FimH (PDB entry 4auu) used as the search model. After several cycles of isotropic temperature-factor refinement, we solved the three-dimensional structure of the FimH–CtbP complex with one molecule in the asymmetric unit. As described previously, CtbP is bound to the N-terminal region of FimH with the mannose ring tightly bound in a hydrophilic pocket able to stabilize the sugar moiety. Indeed, multiple hydrogen bonds are formed to amino acids such as Asn46, Asn135 and Asp140 and also water molecules. Owing to the *trans* stereochemistry of the 1*E* ethene group, the biphenyl part of the ligand accommodates the closed tyrosine gate formed by Tyr48 (χ_1_ = −164.0°, χ_2_ = −169.2°) and Tyr137. The first phenyl ring is stabilized through stacking and T-shaped quadrupolar interactions with Tyr48 and Tyr137, respectively (Fig. 2 ▸ *a*). Moreover, the hydrophobic biphenyl moiety also interacts with the lipophilic amino acids Pro26 and Val27 of a neighbouring FimH molecule because of the crystal packing. Despite the hydrophobic pattern of CtbP, this ligand is solvated with well structured water molecules (average *B* factor of 18.5 Å^2^) on its solvent-exposed side.

### 3.2. Crystal structure of FimH–CcbP   

In order to solve a complete structure of the FimH–CcbP complex, trigonal crystals of native FimH were soaked in ligand solution. In this case, two monomers were present in the asymmetric unit. As for CtbP, the sugar part of the CcbP molecule is located in the same hydrophilic pocket as that observed previously. Nevertheless, because of the new *cis* configuration of the 2*Z* ethene moiety, the biphenyl mannoside is now bound in the open tyrosine gate (Tyr48 side chain χ_1_ = −58.5°, χ_2_ = −89.2°). Furthermore, in one of the two monomers the terminal biphenyl group can interact with the lipophilic amino acid Pro26 and is thus presenting an orientation that is well defined by the electron density. In contrast, in the other monomer the biphenyl group is exposed to bulk solvent and is thus more flexible (Fig. 2 ▸ *b*).

### 3.3. Crystal structure of FimH–CN   

The last structure we refined is the FimH–CN complex, which was also obtained from soaking a FimH crystal. Two monomers were again identified in the asymmetric unit, and the global fold of the monomers was very similar to that obtained for either CtbP or CcbP. However, although the protein remains unchanged, the aglycon part of the ligand is completely different. Since no alkene moiety is present as a linker between the hydrophobic naphthalene group and the hydrophilic sugar, the naphthyl group is totally free to rotate and we clearly observe this phenomenon in the electron-density map. In both monomers the naphthalene moiety is located outside the binding pocket of FimH. Nevertheless, in one monomer (chain *B*) this aromatic group can interact with another naphthyl group and Pro26 thanks to the crystal packing and is thus further stabilized through intramonomer π–π inter­actions with Tyr48 (Fig. 2 ▸ *c*). In the second monomer (chain *A*) the naphthyl group is completely exposed to solvent and hence can adopt various conformations. In this case, we observed a continuum of electron density for the ligand from Ile13 and Phe142 to Tyr48, Ile51 and Tyr137 (Fig. 2 ▸ *d*), certainly showing the highly dynamic aspects of this binding mode. This dynamic aspect is also reflected in the average *B* factor. Because of the high range of motion of the naphthyl group, a conformational change of the Tyr48 side chain from the closed (χ_1_ = −160.2°, χ_2_ = −121.1°) to the opened (χ_1_ = −58.4°, χ_2_ = −95.3°) form of the tyrosine gate occurred. This is in good agreement with the literature, demonstrating the importance of dynamic motion in this region of the lectin (Roos *et al.*, 2013 ▸).

### 3.4. Solvation of *C*-glycosidically linked mannosides   

**Figure 3:**
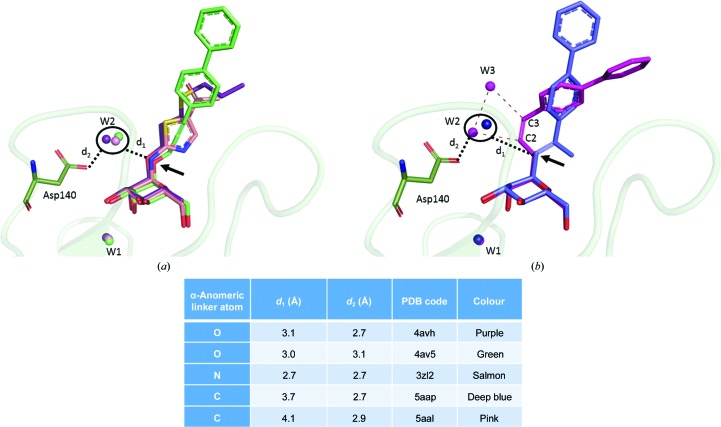
Representation of the water displacement compared with the nature of the α-anomeric linker atom (black arrow). (*a*) Examples of *O*- and *N*-­glycosidically linked mannosides. (*b*) Studied *C*-glycosidically linked mannosides. W1 is a highly conserved water molecule between the 2-OH group of mannose, Phe1 O, Gly14 N and Gln133 OE1, whereas W2 is the water that is displaced upon changing the nature of the atom making the glycosidic linkage to the aglycon substituent. For CcbP (pink) W2 is shifted towards C2 (*d*_W2–C2_ = 3.0 Å). An additional water molecule (W3) interacts strongly with W2 (*d*_W2–W3_ = 3.1 Å) and with C3 (*d*_W3–C3_ = 3.0 Å).

The three structures depicted in the previous sections allowed us to further analyse the effect of the nature of the linker atom on the solvation. We thus decided to compare our new structures with the existing structures of compounds with *O*-glycosidic (PDB entries 4avh and 4av5; Wellens *et al.*, 2012 ▸) or *N*-glycosidic (PDB entry 3zl2; Brument *et al.*, 2013 ▸) linkages. One can observe a highly conserved water molecule (W1) between the 2-OH group of mannose, Phe1 O, Gly14 N and Gln133 OE1. Another water molecule (W2) that forms potential hydrogen bonds to the α-anomeric linker O or N atom is also systematically well conserved in the crystal structures, with a maximal distance of about 3 Å (Fig. 3 ▸ *a*). This second water molecule W2 forms further potential hydrogen bonds to the carboxylate moiety of the Asp140 side chain. In the case of our *C*-glycosidically linked mannosides, water molecule W2 still interacts with Asp140 but has shifted away from the glycosidic linker atom. This can easily be explained by the more pronounced hydrophobic behaviour of *C*-mannoside compounds, which is responsible for the loss of dipolar interactions between water and *O*- or *N*-mannosides. In the case of CcbP, W2 has moved close to the first alkene carbon (C2) (*d*_w2–c2_ = 3.0 Å) and is thus stabilized by the electronic cloud of its π orbitals. In addition, we observed another water molecule (W3) that interacts strongly with W2 (*d*_W2–W3_ = 3.1 Å) and with C3 (d_W3–C3_ = 3.0 Å) (Fig. 3 ▸ *b*). These results underline a totally new hydration pattern that is unique to *C*-linked mannosidic compounds.

## 4. Discussion   

In this study, we highlighted two new features of *C*-mannosylated inhibitors. Firstly, we demonstrated that these new anti-adhesive compounds bind FimH and hence have the potential to serve as potent antagonists of FimH. Indeed, we identified three distinct binding modes related to the stereochemistry of the linkage. CtbP (*trans*) and CcbP (*cis*) can accommodate both open and closed conformations of the tyrosine gate formed by Tyr48 and Tyr137. For the completely saturated compound CN, the binding mode is different from that observed for the unsaturated compound. Because of the high flexibility of the linkage, the aglycon can adopt a wide range of conformations extending between the side chains of Ile13 and Tyr48. Secondly, we proposed a new solvation model for the glycosidic linker to the α-d-mannosides, which exhibits a more hydrophobic behaviour. We found that the linker-associated water molecule is shifted away from the *C*-glycosidic linkages compared with the *O*- and *N*-glycosidic linkages and that another water molecule could come in to assist in hydrogen bonding downstream of the *C*-glycosidic bond. This is in agreement with hydrogen bonds between water and CH being relatively weak compared with nitrogen or oxygen acceptors. This may affect the affinity of the *C*-mannosides for FimH, although they are able to bind to the protein. Further evaluation of the affinities of our new compounds and comparison with known *O*-, *N*- and *S*-glycosidically linked mannosides are planned. These results will help in the design of novel FimH antagonists with a higher therapeutic interest, as carbohydrate-processing enzymes are unable to degrade *C*-glycosidic bonds.

## Supplementary Material

PDB reference: FimH–CcbP, 5aal

PDB reference: FimH–CtbP, 5aap

PDB reference: FimH–CN, 5abz

## Figures and Tables

**Table 1:** Data-collection and refinement statistics for FimH complexes

	FimH–CtbP	FimH–CcbP	FimH–CN
Crystal data			
Space group	*P*2_1_2_1_2_1_	*P*3_1_21	*P*3_1_21
Unit-cell parameters (Å)	*a* = 31.18, *b* = 41.71, *c* = 97.21	*a* = *b* = 90.43, *c* = 79.67	*a* = *b* = 90.83, *c* = 79.87
Subunits per asymmetric unit	1	2	2
Data statistics			
Resolution range (Å)	38.3–1.30	19.8–2.45	39.5–2.40
Unique reflections	26776	14129	15226
Completeness (%)	84.1 (38.3)	99.8 (100)	95.0 (89.4)
*R*_merge_ (%)	6.0 (21.0)	11.0 (36.0)	9.5 (36.0)
〈*I*/σ(*I*)〉	22.9 (7.8)	10.5 (3.8)	14.4 (4.2)
Multiplicity	6.4 (2.5)	3.7 (3.3)	4.0 (3.8)
Refinement			
*R*_cryst_/*R*_free_ (%)	12.4/15.1	13.9/23.0	13.9/20.6
No. of atoms			
Protein	1196	2392	2392
Ligand	26	52	48
Water	271	237	204
Average *B* factors (Å^2^)			
Protein	6.2	22.9	25.4
Ligand	5.9	41.1	36.3
Water	18.6	28.4	30.1
Wilson *B* value (Å^2^)	6.6	22.2	24.7
R.m.s.d.			
Bond lengths (Å)	0.008	0.012	0.012
Bond angles (°)	1.401	1.376	1.432
Ramachandran plot			
Favoured (%)	97.6	96.8	94.9
Outliers (%)	0.0	0.0	0.0
PDB entry	5aap	5aal	5abz
